# Editorial: Advancing neurodevelopmental disorder models with human iPSC and multi-omics integration

**DOI:** 10.3389/fnins.2025.1724360

**Published:** 2025-11-12

**Authors:** Wardiya Afshar-Saber, Vanya Metodieva, Angelica D'Amore

**Affiliations:** 1 Department of Neurology, F.M. Kirby Neurobiology Center, Boston Children's Hospital, Harvard Medical School, Boston, MA, United States; 2 Rosamund Stone Zander Translational Neuroscience Center, Boston, MA, United States; 3 University of St Andrews, St Andrews, United Kingdom

**Keywords:** disease modeling, optogenetics, multi-omics, neurodevelopmental disorders, induced pluripotent stem cells (iPSCs)

Neurodevelopmental disorders (NDDs) are a highly heterogeneous group of diseases that impair children's social, cognitive, and emotional functioning (D'Amore et al., 2023; Khodosevich and Sellgren, 2023). Despite their diversity, these disorders share a common obstacle: the lack of experimental systems that faithfully recapitulate human neurodevelopment and its perturbations. For decades, progress has been constrained by the limitations of animal models, which, although invaluable, do not capture the intricacies of human-specific developmental processes (Zhao and Bhattacharyya, 2018). In this context, the emergence of human induced pluripotent stem cells (hiPSCs) has opened a new era, enabling the derivation of patient-specific neurons, glia, and generation of three-dimensional (3D) organoid systems that more closely model human physiology and pathology.

The integration of hiPSC technology with advanced functional assays, imaging platforms, and multi-omics approaches has helped unravel the cellular and molecular mechanisms underlying NDDs. However, quantifying the balance of neuronal excitation and inhibition, deciphering transcriptional and epigenetic dysregulation, and resolving abnormalities in protein trafficking and chromatin structure remain challenging. In this Research Topic, we present a collection of four original research articles and two reviews illustrating how diverse, yet complementary strategies are being deployed to overcome these challenges, offering both mechanistic insights and translational opportunities.

## Disease modeling and mechanistic discovery

One of the greatest strengths of hiPSC-based models lies in their capacity to retain the genetic background of the patient, thus providing direct access to disease mechanisms (Takahashi et al., 2007; Yu et al., 2007). In this original research article, Wu et al. demonstrate this principle in the context of epilepsy by generating iPSCs from patients harboring a novel *CLCNKB* mutation. By differentiating these cells into neurons and performing transcriptomic profiling, they identify differentially expressed genes implicated in epileptogenesis, thereby uncovering gene networks that may contribute to seizure susceptibility. Similarly, studies of Tuberous Sclerosis Complex (TSC) have leveraged hiPSC-derived neurons to interrogate dysregulation of the mTOR signaling pathway (Winden et al., 2019; Afshar Saber and Sahin, 2020). In this original research article, Buttermore et al. show that newly developed mTORC1-selective inhibitors rescue hyperexcitability and abnormal neuronal morphology in *TSC*2^−/−^ neurons, pointing to novel therapeutic strategies. Collectively, these works demonstrate how hiPSC models can serve as powerful platforms for mechanistic discovery as well as for preclinical drug testing.

## Organoid systems for developmental complexity

Beyond two-dimensional (2D) cultures, 3D brain organoids are increasingly used to capture and model more complex features of neurodevelopment (Lancaster et al., 2013; Paşca et al., 2015). In this review article, Winden et al. emphasize the power of organoid systems for studying malformations of cortical development, conditions that often present with microcephaly, disorganized placement of cell types, and severe cognitive impairments. By recapitulating important aspects of early developmental processes such as progenitor proliferation, neuronal migration, and layer formation, organoids allow the identification of disrupted pathways, from cytoskeletal regulation to growth factor signaling that drive cortical malformations. Therefore, brain organoids represent an advanced platform that recapitulates phenotypic and mechanistic features of human cortical development, which are inadequately modeled in animal systems.

## Integrating multi-omics and computational frameworks

As experimental systems become more sophisticated, they generate increasingly complex datasets spanning genomics, transcriptomics, proteomics, and metabolomics (Fleck et al., 2023). The challenge is no longer only how to collect such data, but also how to integrate and interpret it meaningfully (Pinu et al., 2019). In this original research article, Lichtarge et al. introduce *MetaboLINK*, a computational tool that combines principal component analysis with graphical lasso to parse longitudinal metabolomics data. When applied to differentiating neural cells, the method reveals stage-specific metabolic programs involving amino acids, lipids, and energy utilization, providing insight into how metabolism shapes neurodevelopment. This work illustrates the power of computational innovations to connect high-dimensional data with biological processes, thereby enhancing the interpretability and utility of multi-omics approaches in NDD research.

## Functional phenotyping, machine learning, and biomarker discovery

hiPSC-derived models provide not only molecular but also functional readouts, including electrophysiological recordings, multielectrode array analyses and calcium imaging (Afshar-Saber et al., 2024). Yang et al. review how these rich datasets can be combined with machine learning to classify subtle phenotypic signatures, accelerate drug screening, and improve disease modeling in both 2D cultures and 3D organoids. Such integration of experimental and computational approaches exemplifies the field's shift toward predictive and data-driven frameworks. At the same time, the translational reach of neurological research is expanding beyond cellular modeling to encompass biomarker discovery. In this original research article, Ashok et al. identify lectin-type oxidized LDL receptor-1 (LOX-1) as a promising candidate for monitoring cerebral cavernous malformations (CCM), reporting elevated levels in both urine samples and lesional tissue. By demonstrating the feasibility of detecting disease-associated molecules in non-invasive biospecimens, this work illustrates advances in both mechanistic insight and clinical application.

## Outlook

Taken together, the contributions to this Research Topic highlight a multidimensional research landscape in which hiPSC-derived neuronal cultures and 3D brain organoid systems are paired with advanced functional imaging, and multi-omics to probe the pathophysiology of NDDs. Patient-specific hiPSCs enable mechanistic discovery in disorders such as epilepsy and TSC, while cortical organoids provide a unique window into the origins of structural brain malformations. Multi-omics tools such as *MetaboLINK* and machine learning–driven phenotyping underscore the importance of computational innovation for extracting insights from complex datasets, while biomarker discovery efforts, as exemplified by LOX-1 in CCM, extend the translational scope of this work into diagnostics and therapeutic monitoring.

Despite this progress, significant challenges remain. Standardization of differentiation protocols (Anderson et al., 2021), reproducibility across laboratories (Glass et al., 2024; Sandoval et al., 2024), and integration of massive multi-layered datasets continue to be pressing issues. Moreover, translating findings from cellular and molecular models into clinical interventions will require close collaboration between basic scientists, clinicians, and computational experts. Nevertheless, the trajectory is clear: by uniting stem cell biology, multi-omics integration, and computational frameworks, the field is moving toward more predictive, patient-specific, and ultimately actionable models of neurodevelopmental disorders.

This Research Topic not only captures the state of the art but also illuminates a path forward by demonstrating how methodological innovation and interdisciplinary collaboration can collectively overcome the limitations of earlier models and bring the promise of therapies and diagnostics closer to realization.
